# Protective Effects of Orange Sweet Pepper Juices Prepared by High-Speed Blender and Low-Speed Masticating Juicer against UVB-induced Skin Damage in SKH-1 Hairless Mice

**DOI:** 10.3390/molecules27196394

**Published:** 2022-09-27

**Authors:** Van-Long Truong, Razanamanana. H. G. Rarison, Woo-Sik Jeong

**Affiliations:** Food and Bio-Industry Research Institute, School of Food Science and Biotechnology, College of Agriculture and Life Sciences, Kyungpook National University, Daegu 41566, Korea

**Keywords:** high-speed blender, low-speed masticating juicer, skin health, sweet pepper juice, UVB radiation

## Abstract

Sweet pepper fruits (*Capsicum annuum* L.) contain various nutrients and phytochemicals that enhance human health and prevent the pathogenesis of certain diseases. Here, we report that oral administration of orange sweet pepper juices prepared by a high-speed blender and low-speed masticating juicer reduces UVB-induced skin damage in SKH-1 hairless mice. Sweet pepper juices reduced UVB-induced skin photoaging by the regulation of genes involved in dermal matrix production and maintenance such as collagen type I α 1 and matrix metalloproteinase-2, 3, 9. Administration of sweet pepper juices also restored total collagen levels in UVB-exposed mice. In addition, sweet pepper juices downregulated the expression of pro-inflammatory proteins such as cyclooxygenase-2, interleukin (IL)-1β, IL-17, and IL-23, which was likely via inhibiting the NF-κB pathway. Moreover, primary antioxidant enzymes in the skin were enhanced by oral supplementation of sweet pepper juices, as evidenced by increased expression of catalase, glutathione peroxidase, and superoxide dismutase-2. Immunohistochemical staining showed that sweet pepper juices reduced UVB-induced DNA damage by preventing 8-OHdG formation. These results suggest that sweet pepper juices may offer a protective effect against photoaging by inhibiting the breakdown of dermal matrix, inflammatory response, and DNA damage as well as enhancing antioxidant defense, which leads to an overall reduction in skin damage.

## 1. Introduction

The skin, the largest organ of the human body, is composed of the epidermis, dermis, and subcutis, which provide the first line of defense against external environmental factors, such as physical damage, temperature shock, pathogens, and ultraviolet (UV) radiation [1]. In addition, skin appearance, especially human facial skin, importantly contributes to self-confidence, social interaction, and quality of life. Various risk factors, including genetics, hormonal changes, metabolic processes, and environmental stimuli, such as UV radiation, xenobiotics, and bacteria, can affect skin structure, function, and appearance [2].

Sunlight is one of the most crucial causes of skin aging, also known as photoaging, and accounts for up to 90% of visible skin aging [3]. Skin photoaging is characterized by mottled pigmentation, roughness, dryness, visible wrinkles, and decreased elasticity. Although UVB accounts for only a small portion of total UV radiation, it can penetrate the basal cell layer of epidermal cells and causes serious damage to the epidermis and dermis of the skin and is therefore primarily responsible for sunburn and erythema [3,4]. In addition, UVB also provokes the degradation of extracellular matrix (ECM) composition in the dermis, which is composed primarily of type I collagen with lesser amounts of type II collagen, elastin, proteoglycans, and fibronectin, thereby leading to the loss of structure and integrity, laxity, and wrinkles [4,5].

Numerous epidemiological and laboratory studies suggest regular consumption of fruits and vegetables can enhance skin health and delay skin aging [6]. A wide range of phytochemicals stemming from edible plants has been reported to exert antioxidant and anti-inflammatory activities, which contribute to alleviating inflammatory response, restoring the functionality of the skin from photodamage, and preventing further progression of solar UV-induced skin disorders [7]. Sweet peppers, belonging to the genus *Capsicum* with more than 200 varieties, are considered natural sources of bioactive compounds such as carotenoids, vitamin C and E, flavonoids, phenolic compounds, and dietary fiber [8]. Previous studies alongside research in our laboratory found that orange sweet pepper fruit contains a wide range of carotenoids, such as viola, anthera, capsanthin, lutein, zeaxanthin, cryptoxanthin, carotene, and lycopene, in which lutein, β-cryptoxanthin, α-carotene, and zeaxanthin contents are higher than those in other sweet pepper fruits [9,10,11]. From the pharmaceutical point of view, sweet pepper exerts various health benefits, such as antioxidant [12], anti-inflammation [13], cancer chemopreventive activity [14], and prevention of cardiovascular diseases [15]. Yellow/orange/red sweet pepper fruit extracts were found to inhibit the lipopolysaccharide-induced inflammatory response by inducing heme oxygenase (HO)-1 in RAW 264.7 macrophages [16]. Using in vitro and in vivo models, fermented yellow/orange/red sweet pepper fruits with *Lactobacillus plantarum* have been indicated to exert protective effects against oxidative stress-mediated retinal damage [17]. Four colors of sweet peppers (green, yellow, orange, and red) exert antioxidant activities and inhibitory effects on Alzheimer’s disease-associated key enzymes, such as acetylcholinesterase, butyrylcholinesterase, and β-secretase, and thereby may be useful for the prevention of Alzheimer’s disease [18]. In addition, clinical studies have shown that the administration of red sweet pepper xanthophylls inhibited UV-induced skin damage [19] and improved moisture on facial skin in volunteers [20].

Fresh vegetable/fruit juice has been receiving great interest from consumers because of a simple household juicing preparation, retaining the major health-promoting components of whole vegetables/fruit, and the absence of additives and preservatives [21,22]. Consequently, household juicers based on several technologies such as centrifugal blending and cold-pressing were developed [22]. Although physicochemical characteristics of vegetable/fruit juice prepared by several different household juicers have been evaluated, the effects of juicing methods on the biological activity of the juice in experimental models have seldomly been investigated [23,24]. Therefore, this study aimed to evaluate and compare the photoprotective effects of fresh orange sweet pepper juices prepared by a household high-speed blender (HSB) and a low-speed masticating juicer (LSM) against UVB-induced skin damage in SKH-1 hairless mice.

## 2. Results and Discussion

### 2.1. HSB and LSM Sweet Pepper Juices Inhibit UVB-Induced Photoaging in SKH-1 Hairless Mice

The consumption of fresh vegetable/fruit juice has recently been attracting great interest in the improvement of skin health. Fresh juice contains a wide range of bioactive compounds, such as carotenoids and polyphenols that contribute to maintaining skin functions and preventing skin injury and carcinogenesis. Carotenoids have been demonstrated to exert various benefits on the skin, including protection against sunlight-induced erythema, pigmentation and collagen degradation, the inhibition of UV-induced oxidative stress and inflammation, and the prevention of photoaging and photocarcinogenesis [25]. In addition, amounting studies have indicated that carotenoid supplementation for 3–24 weeks increases serum levels of carotene, lutein, and lycopene, as well as skin levels of β-carotene and total carotenoids [26,27,28,29]. Furthermore, dietary carotenoids such as α/β-carotene, lutein, and zeaxanthin are distributed to various layers of skin and the highest levels are often found in the stratum corneum closest to the skin surface to protect skin from harmful effects of sunlight [25,30]. On the other hand, UV radiation could reduce the levels of β-carotene, lycopene, and other carotenoids in the skin, which may potentially be attributed to ROS, thereby increasing the risk of photodamage [31,32]. Taken together, regular intake of carotenoid-rich vegetables/fruits may be an effective strategy for skin health enhancement as well as complementary protection against solar UV-induced skin damage.

**Figure 1:**
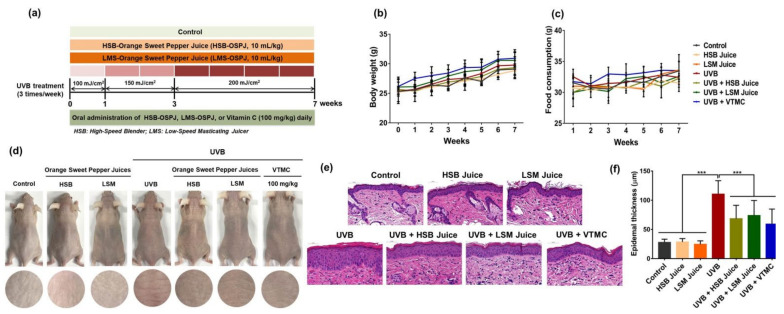
Protective effects of HSB and LSM sweet pepper juices against photoaging in UVB-irradiated SKH-1 hairless mice. (**a**) Animal experimental design. (**b**) Body weight. (**c**) Food consumption. (**d**) Photos of dorsal mouse skin. (**e**) Representative images of H&E staining. (**f**) Epidermal thickness. Data are expressed as the mean ±SD. *** *p* < 0.001 values are considered as statistically significant differences. HSB, high-speed blender; LSM, low-speed juicer; VTMC, vitamin C.

Protective effects of HSB and LSM sweet pepper juices against photoaging were examined using UVB-irradiated hairless mice and animal experimental design was illustrated in Figure 1a. Results showed that no significant difference in body weight and food consumption among experimental groups was observed throughout 7 weeks of treatment, suggesting that sweet pepper juices did not exhibit adverse effects (Figure 1b,c). Compared to the normal control, HSB, or LSM juice-only group, skin wrinkle was obviously observed in UVB-irradiated mice, whereas wrinkle development was suppressed by oral administration of sweet pepper juices (Figure 1d). In addition, H&E staining revealed a considerable increase in epidermal thickness in UVB-irradiated mice (Figure 1e,f). However, oral administration of sweet pepper juices significantly decreased epidermal hyperplasia and no significant difference among HSB juice, LSM juice, and positive control vitamin C groups was observed. This observation is in line with previous studies, which suggest that the hyperplasia of the epidermis in hairless mice irradiated with UV radiation is associated with the hyperproliferation of keratinocytes [33,34]. In normal human and mouse skin, the apoptotic and proliferative processes of keratinocytes are tightly regulated to construct the epidermis and chronic UV radiation may dysregulate these two events, resulting in the proliferation of keratinocytes to replace apoptotic cells and subsequently leading to the development of skin cancer [35,36].

**Figure 2:**
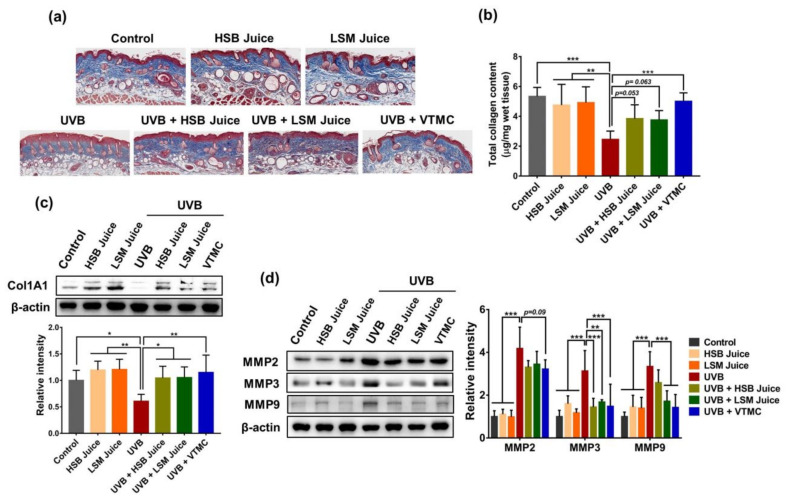
Effects of HSB and LSM sweet pepper juices collagen content in UVB-irradiated SKH-1 hairless mice. (**a**) Representative images of Masson’s trichrome stain for collagen fibber (Blue); (**b**) Total collagen content in skin tissues from each group; (**c**) Protein expression of Col1A1 in skin tissues; (**d**) Protein expression of matrix proteinase (MMP)-2, MMP3, and MMP-9 in skin tissues. Data are expressed as the mean ±SD. * *p* < 0.05, ** *p* < 0.01, and *** *p* < 0.001 values are considered as statistically significant differences. HSB, high-speed blender; LSM, low-speed juicer; VTMC, vitamin C.

Skin photoaging is characterized by the atrophy of the dermal connective tissue that is associated with the destruction of extracellular matrix (ECM) components, particularly collagen fibers. UV radiation stimulates the synthesis of matrix metalloproteinase (MMP) members, such as MMP-1, -2, -3, -9, and -13 in the skin keratinocytes and fibroblasts, which in turn degrade collagen and other ECM proteins, thereby leading to wrinkle formation and skin aging [37]. Increased levels of MMPs are found in murine and human skins irradiated with UV radiation [5,38]. In addition, UV radiation also reduces collagen production, mainly through downregulation of types I and III procollagen synthesis [39]. This study confirmed that UVB considerably decreased collagen content in skin tissues, as indicated by Masson’s trichrome staining and total collagen content assay (Figure 2a,b). However, oral supplementation of HSB or LSM juice partially restored the level of collagen fiber. Consistently, UVB significantly downregulated the expression of collagen type I alpha 1 (Col1A1) but upregulated the expression of matrix collagen degradation enzymes such as MMP-2, MMP-3, and MMP-9 (Figure 2c,d). Such effects were reversed by the daily consumption of sweet pepper juices. A recent study has shown that carotenoids- and polyphenol-rich extracts reduced MMP-1 expression and simultaneously enhanced procollagen synthesis in normal human dermal fibroblasts under the condition of oxidative stress [40]. Although both HSB and LSM juices did not reduce MMP-2 expression and LSM juice was more effective in reducing MMP-9 expression, in general, the efficacy of HSB and LSM juices was similar and comparable to that of positive control vitamin C. These findings suggest that sweet pepper juice exerts anti-photoaging activity in a model of UVB-irradiated hairless mice by increasing collagen synthesis and suppressing collagen degradation.

### 2.2. HSB and LSM Sweet Pepper Juices Attenuate Inflammatory Response in UVB-Irradiated SKH-1 Hairless Mice

Chronic UVB irradiation is reported to cause skin inflammation, as characterized by accelerating the production of pro-inflammatory mediators and cytokines. Cyclooxygenase-2 (COX2) catalyzes the rate-limiting step in the conversion of arachidonic acid into prostaglandins [41]. UV-induced COX-2 expression stimulates keratinocyte proliferation and epidermal hyperplasia, induces the production of other pro-inflammatory mediators, and triggers skin carcinogenesis [42,43]. Furthermore, COX-2 is also recognized as a molecular link between inflammation and tumorigenesis [44]. Transgenic SKH-1 mice with COX-2 overexpression promote the development of UV-induced skin carcinogenesis, whereas COX-2 knockout mice exhibit significant mitigation of UV-elicited skin tumorigenesis [43,45]. In addition, selective COX-2 inhibitors were found to considerably suppress UV-induced epidermal hyperplasia and inflammation, as well as skin tumorigenesis in terms of tumor number and tumor size [42,46,47]. Therefore, inhibition of COX-2 expression may be a beneficial strategy for preventing UV-caused skin injury and carcinogenesis.

**Figure 3:**
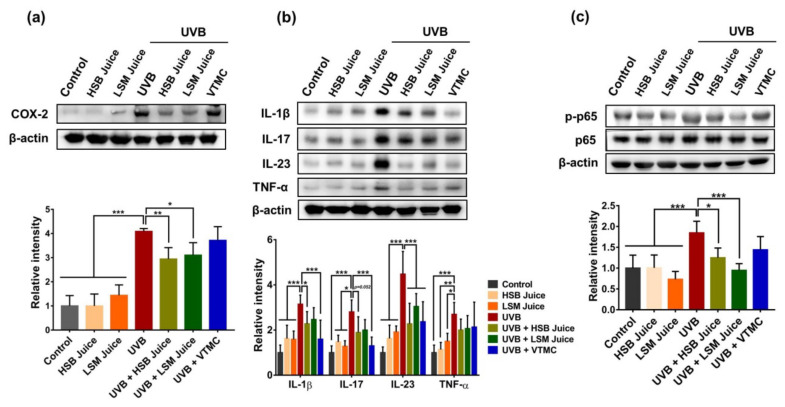
Anti-inflammatory effects of HSB and LSM sweet pepper juices in UVB-irradiated SKH-1 hairless mice. (**a**) Protein expression of COX-2; (**b**) Protein expression of IL-1β, IL-17, IL-23, and TNF-α in skin tissues; (**c**) Protein expression of p-p65 and p65 in skin tissues. Data are expressed as the mean ±SD. * *p* < 0.05, ** *p* < 0.01, and *** *p* < 0.001 values are considered as statistically significant differences. HSB, high-speed blender; LSM, low-speed juicer; VTMC, vitamin C.

Aberrant expression of COX-2 was observed in the models of human and murine skins acutely and chronically irradiated with UVB [48,49,50]. In the present study, chronic exposure of mice to UVB remarkably induced COX-2 expression in skin tissues (Figure 3a). However, UVB-induced COX-2 expression was significantly decreased by daily consumption of sweet pepper juices. In addition, under the condition of UV irradiation, keratinocytes and other skin-associated cells secrete a large number of pro-inflammatory cytokines, such as interleukin (IL)-1β, IL-6, IL-17, IL-23, and tumor necrosis factor (TNF)-α that play a crucial role in UV-induced skin inflammation due to their action of inducing edema and erythema, decreasing skin integrity, and increasing chemoattractant and other inflammatory molecules expression and ROS formation [51,52]. Moreover, these pro-inflammatory cytokines also provoke massive infiltration of immune cells, especially neutrophils that excrete matrix metalloproteinases capable of degrading extracellular matrix components and produce ROS likely to devastate vital cellular components and thereby enhance UV-induced skin damage [53,54]. Considering the suppression of pro-inflammatory cytokine production may contribute to the activity of sweet pepper juices, the levels of IL-1β, IL-23, IL-17, and TNF-α were examined. Results indicated that HSB and LSM sweet pepper juices significantly reduced protein levels of IL-1β and IL-23, but not IL-17 and TNF-α in UVB-irradiated mice (Figure 3b). Such inhibition of inflammatory factors partially explains the ability of sweet pepper juice in reducing UVB radiation-induced skin edema and inflammation, epidermal thickness, and skin aging. On the other hand, no significant difference in anti-inflammatory activity between HSB- and LSM-juices was observed, suggesting that the efficiency of fresh sweet pepper juice was independent to juicing methods.

Nuclear factor kappa (NF-κB) is a redox-sensitive transcription factor responsible for regulating the transcriptional expression of a wide range of pro-inflammatory factors, such as COX-2, cytokines, chemokines, and adhesion molecules [55]. Activation of NF- κB pathway by UV radiation is observed in many models of murine and human skins [56,57,58,59]. Thus, we further explored effects of HSB and LSM juices on NF-κB signaling pathway in UVB-exposed mice. Results showed that oral administration of sweet pepper juices significantly inhibited UVB-induced NF-κB activation, as evident from decreased p65 phosphorylation (Figure 3c). Although there was not significant difference between HSB and LSM juices, LSM juice appeared to exert more pronounced anti-inflammatory activity than HSB juice. In particular, the anti-inflammatory effect of sweet pepper juices seemed to be better than that of vitamin C. These data suggest that sweet pepper juices may inhibit UVB-induced skin inflammation by suppressing NF-κB pathway.

### 2.3. HSB and LSM Sweet Pepper Juices Enhance Antioxidant Defense Systems in UVB-Irradiated SKH-1 Hairless Mice

The skin is one of a few organs that come into direct contact with exogenous oxidative sources, such as sunlight. Solar UV radiation can promote the formation of reactive oxygen species (ROS) such as superoxide anion radical, hydrogen peroxides, hydroxyl radicals, and other oxidants that directly and/or indirectly damage to biomolecules, such as proteins, lipids, and nucleic acids and interrupts cellular signaling pathways, eventually leading to skin damage. Approximately 80% of ROS in the skin are thought to be produced by UVA and UVB irradiation [60].

Oxidative stress is thought to play a central role in triggering and driving the signaling events that lead to cellular responses, such as cell death, cellular senescence, and inflammation, following UVB radiation. ROS can activate redox-sensitive transcription factors, such as NF-κB and AP-1 that upregulate the production of inflammatory mediators and cytokines. In addition, UVB-induced ROS also stimulates the release of MMP enzymes, such as MMP-1, -2, -3, -9, and -13, in keratinocytes and fibroblasts, which in turn degrade collagen and other ECM proteins, and simultaneously attenuate collagen synthesis in the skin [1,61]. Therefore, oxidative stress mediates a variety of UVB radiation-induced skin damage such as edema, erythema, hyperplasia, inflammation, and skin aging through inappropriate regulation of signaling pathways, thereby increasing the risk of skin cancer.

Skin cells are equipped with an antioxidant defense system, consisting of enzymatic and non-enzymatic antioxidants, which maintain the pro-oxidant/antioxidant balance by ROS elimination. Nevertheless, flooding of ROS can overwhelm the cellular antioxidant defense capacity and further generate reactive oxidants, resulting in oxidative stress and consequently oxidative photodamaging of the skin [62]. Therefore, regular supplementation of antioxidants is a useful strategy to prevent adverse effects of solar UV radiation.

A battery of antioxidant enzymes plays a vital role in counteracting excessive ROS accumulation, thereby maintaining cellular redox homeostasis. Primary antioxidant enzymes, including catalase (CAT), glutathione peroxidase (GPx), and superoxide dismutase (SOD) are considered to be the most important antioxidant defense in the skin [62]. SOD acts as the first line of defense that catalyzes the dismutation of superoxide anion into oxygen and hydrogen peroxide, with the latter being further decomposed by CAT and GPx [63]. Earlier studies reported that chronic UV exposure reduced protein levels of activities of primary antioxidant enzymes, resulting in a wide range of skin disorders such as sunburn, erythema, photoaging, and even cancer [64,65,66].

**Figure 4:**
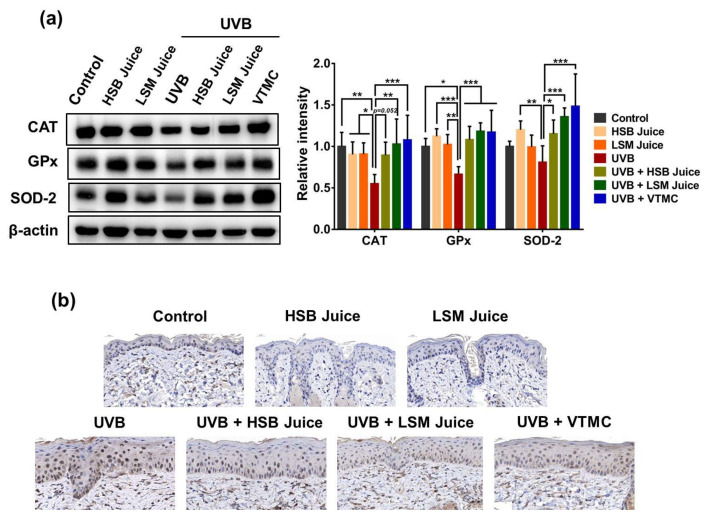
Antioxidant effects of HSB and LSM sweet pepper juices in UVB-irradiated SKH-1 hairless mice. (**a**) Protein expression of CAT, GPx, and SOD-2 in skin tissues; (**b**) Immunostaining for 8-OHdG in skin tissues. Data are expressed as the mean ±SD. * *p* < 0.05, ** *p* < 0.01, and *** *p* < 0.001 values are considered as statistically significant differences. HSB, high-speed blender; LSM, low-speed juicer; VTMC, vitamin C.

To further investigate whether the protective effects of HSB and LSM sweet pepper juices were linked to the enhancement of the antioxidant defense system, we examined the expression of antioxidant enzymes in the mouse skin tissues. UVB irradiation abolished the expression of primary antioxidant enzymes, including CAT, GPx, and SOD-2, suggesting provoked oxidative stress in UVB-exposed mouse skin (Figure 4a). In contrast, both HSB and LSM juices significantly restored the levels of these antioxidant enzymes. Although there was not a significant difference between HSB and LSM juices, LSM juice appeared to be more effective in inducing the expression of antioxidant enzymes than HSB juice.

DNA damage induced by UVB irradiation occurs mainly via oxidative processes and 8-OHdG is the major mutagenic form of oxidative DNA damage [67]. The present study hypothesizes that sweet pepper juice acts as an antioxidant capable of protecting cells/tissues from DNA damage caused by UVB radiation. Immunohistochemical analysis indicated a high level of 8-OHdG in the mouse skin chronically irradiated with UVB radiation, compared to the normal control, HSB, or LSM juice-only group. However, both HSB and LSM sweet pepper juices exerted a protective effect against UVB-induced DNA damage, as indicated by a reduced level of 8-OHdG (Figure 4b). Taken together, these findings suggest that sweet pepper juice exerts antioxidant activity to protect against UVB-induced skin injury along with its anti-inflammatory property.

## 3. Conclusions

In conclusion, the regular intake of fresh vegetable/fruit juice containing various intact phytonutrients without preservatives is believed to bring about health benefits, including skin health improvement. Sweet orange pepper fruit with high levels of carotenoids, vitamin C, and other bioactive components have the potential for use in a dietary supplement for skin health. In this study, oral administration of sweet pepper juice exerted a protective effect against UVB-induced skin aging in a hairless mouse model by inhibiting MMP expression and restoring collagen synthesis, thereby preventing skin wrinkles. UVB-induced skin inflammation was suppressed by the consumption of sweet pepper juice. Simultaneously, sweet pepper juice also maintained the antioxidant defense capacity of the skin and thus prevented UVB-induced oxidative damage. In addition, juicing methods were considered in the present study and sweet pepper juice prepared by an LSM juicer may exhibit pronounced efficacies, compared to the juice by HSB. However, different experimental models with long-term consumption are required to compare the biological effects of HSB and LSM juices.

## 4. Materials and Methods

### 4.1. Materials

Formalin, hematoxylin, and eosin were purchased from Sigma-Aldrich (St. Louis, MO, USA). Anti-Col1A1, p-p65, p65, COX-2, MMP-2, MMP-3, TNF-α, and peroxidase-conjugated secondary anti-rabbit antibodies were obtained from Cell Signaling Technology (Boston, MA, USA). Anti-catalase, GPx, SOD-2, IL-1β, Il-17, Il-23, MMP-9, and peroxidase-conjugated secondary anti-mouse antibodies were purchased from Santa Cruz Biotechnology (Santa Cruz, CA, USA). Anti-8-OHdG antibody was procured from Thermo Scientific (Waltham, MA, USA). All other chemicals used in this study were of analytic grade.

### 4.2. Preparation of Orange Sweet Pepper Juice

Orange sweet pepper fruits (*Capsicum annuum* L.) were purchased from Hoengseong Rainbow farm (Gangwon-do, South Korea) and processed right after arrival. For purpose of this study, fresh orange sweet pepper juices were prepared daily using a high-speed blender (HSB, HC-BL 2000, HappyCall, Gimhae, South Korea) and a low-speed masticating juicer (LSM, H200-DBFA03, Hurom Co. Ltd. Gimhae, South Korea). The major bioactive constituents in orange sweet pepper juices were analyzed. Total phenolic content in LSM and HSN juices was 111.77 ± 1.88 mg/100 mL and 96.45 ± 3.56 mg/100 mL, respectively, while total carotenoid content was 3.49 ± 0.01 mg/100 mL and 4.18 ± 0.1 mg/100 mL, respectively. The level of total ascorbic acid was 119.80 ± 3.19 mg/100 mL for LSM juice and 100.63 ± 1.10 mg/100 mL for HSB juice.

### 4.3. Animal Experimental Design and UVB Irradiation

Six-week-old female SKH-1 hairless mice were purchased from Orient Bio (Seongnam, South Korea) and housed in plastic cages at 25 ± 2 °C and 50 ± 5% relative humidity under a 12–12 h light–dark cycle. All animals were allowed free access to standard food and water ad libitum. All experiment protocols were approved by the Institutional Animal Care and Use Committee of Kyungpook National University (No. KNU 2021-182). After a week of acclimation, mice were randomly divided into seven groups (n = 10): Normal control, HSB juice (10 mL/kg), LSM juice (10 mL/kg), UVB, UVB + HSB juice (10 mL/kg), UVB + LSM juice (10 mL/kg), and UVB + Vitamin C (positive control, 100 mg/kg). HSB juice, LSM juice, or Vitamin C were orally administered to the animals in each group daily for 7 weeks, while the mice in the normal control and UVB groups were given an equal amount of distilled water. Vitamin C is well-known to protect against UVB-induced skin damage [68,69] and vitamin C (100 mg/kg) was used as a positive control according to previous studies [70,71]. Food intake and body weight were regularly recorded throughout the study. UVB irradiation was performed three times per week for 7 weeks using a BioLink Crosslinker system (Vilber Lourmat, Paris, France) with peak emission at 312 nm. The UVB dose was started at 100 mJ/cm^2^ in the first week and 150 mJ/cm^2^ in the following two weeks and increased up to 200 mJ/cm^2^ in the last four weeks. At the end of the experiment, the mice were sacrificed with CO_2_ and dorsal skin tissues were rapidly harvested. After removing lipid and connective tissues, a part of skin tissues was cut and fixed in a 10% formalin solution, and the rest of the tissue samples were snap-frozen in liquid nitrogen and then stored at −80 °C for further analysis.

### 4.4. Histological and Immunohistochemical Analyses

Dorsal skin tissues of each group were fixed in 10% neutral formalin and embedded in paraffin. Sections were stained with hematoxylin and eosin (H&E) for general histopathology and Masson’s trichrome to examine epidermal thickness and collagen fiber, respectively. For immunostaining, the sections were incubated with specific mouse anti-8-OHdG primary antibody in a humidified chamber overnight at 4 °C and followed by incubation with horseradish-conjugated secondary antibody and 3,3’-diaminobenzidine. Representative H&E, Masson’s trichrome, and immunostained images of sections were captured using a digital microscope (Paxcam, Villa Park, IL, USA).

### 4.5. Measurement of Total Collagen Content

Dorsal skin tissues were homogenized in distilled water and total collagen content was measured by total collagen assay kit (Biovision, Waltham, MA, USA) according to the manufacturer’s instructions.

### 4.6. Western Blot Analysis

Dorsal skin tissues were homogenized in RIPA buffer containing protease and phosphatase inhibitors (Thermo Fisher Scientific, Waltham, MA, USA) using Precellys 14 tissue homogenizer (Bertin Technologies, Montigny-le-Bretonneux, France). The lysates were then centrifuged at 13,000× *g* for 15 min at 4 °C to obtain supernatant. Protein samples were aliquoted out and stored at −80 °C. The protein concentrations were measured using a PierceTM BAC protein assay kit (Thermo Fisher Scientific). An equal amount of protein samples was resolved on SDS-PAGE gel and then transferred to polyvinylidene fluoride membrane using a semidry transfer system (Bio-rad, Hercules, CA, USA). After blocking with 5% non-fat skim milk, the membranes were incubated with specific primary antibodies overnight at 4 °C and followed by hybridization with proper secondary antibodies for 3 h at 4 °C. Eventually, protein bands were visualized using Western Blotting Luminol reagent (Santa Cruz Biotechnology).

### 4.7. Statistical Analysis

Data are presented as the mean ± standard deviation (SD) of at least three independent experiments. Statistical analysis was performed using analysis of variance followed by the Tukey’s post hoc test. The *p*-value < 0.05 was considered to be statistically significant.

## Data Availability

Not applicable.

## Sample Availability

Not available.
